# The nucleus accumbens: a target for deep brain stimulation in resistant major depressive disorder

**DOI:** 10.1186/2049-9256-1-17

**Published:** 2013-10-23

**Authors:** Cecilia Nauczyciel, Suzanne Robic, Thibaut Dondaine, Marc Verin, Gabriel Robert, Dominique Drapier, Florian Naudet, Bruno Millet

**Affiliations:** Academic Department of Adult Psychiatry, Guillaume Régnier Hospital, EA 4712 35000 Rennes, France; Lyon Neuroscience Research Center INSERM U1028 - CNRS UMR 5292 Dynamics and Brain Cognition, 69675 Bron, France; Rennes University Hospital Centre Research Unit EA 4712 Behavior and Basal Ganglia, 35000 Rennes, France

**Keywords:** Major depressive disorder, Deep brain stimulation, Dopamine, Nucleus accumbens

## Abstract

**Objective:**

This review aimed to investigate the therapeutic potential of Deep Brain Stimulation (DBS) for treating resistant Major Depressive Disorder (MDD). We explored the role of Nucleus accumbens (Nac) as a target for treatment.

**Method:**

We made a systematic review of all studies examining the mechanisms of action of high frequency brain stimulation and the pathophysiology of MDD. We also reported all the studies exploring the therapeutic potential of DBS in MDD.

**Results:**

As a central relay-structure, the Nac seems to play a central role in MDD symptomatology. We investigated its role as a primary target for DBS in depressed patients. Anatomically the Nac is at the centre of the interactions between dopaminergic, serotoninergic and glutamatergic systems. Functionally, the Nac is involved in both normal and abnormal reward processes and in anhedonia and loss of motivation. Due to its central location between the emotional system, the cognitive system and motor control system, the Nac seems to have a central role in mood and feeling regulation.

**Conclusion:**

According to encouraging recent studies, DBS seems to be a promising technique in resistant MDD treatment.

## Review

### Introduction

Major depressive disorder (MDD) is a common psychiatric mood disorder. MDD consists of a single episode or several instances of recurrent or relapsed episodes of major depression. According to the DSM IV [1] major depression is characterized by depressed mood or loss of interest in daily activities. These symptoms can be associated with physical manifestations such as: significant weight loss or weight gain, insomnia or hypersomnia, diminished ability to think or concentrate and fatigue or loss of energy. A feeling of worthlessness, excessive or inappropriate guilt and recurrent thoughts of death can also been reported. The symptoms cause clinically significant distress or impairment in social, occupational, or other important areas of functioning. MDD is often associated with a high level of morbidity and mortality and represents the most important risk factor for suicide on the whole life (attributable risk of 28% [2]). Although psychopharmacological treatments are effective for many patients, most of the short-term studies indicate that 29% to 46% of depressed patients remain resistant to treatment [3]. Given the important negative impact on public health of MDD, and data suggesting only modest effectiveness of existing psychological and pharmacological treatments for chronic Treatment Resistant Depression (TRD), there has been increasing interest in exploring the therapeutic potential of non-pharmacological interventions, such as Deep Brain Stimulation (DBS). Recent advances in stereotaxic neurosurgical methods have provided an innovative and promising technique for reducing MDD symptoms in treatment-resistant patients.

Place of DBS in brain stimulation treatments of depression.

Repetitive Transcranial Magnetic Stimulation (rTMS) is a more recent technique of non-invasive stimulation approved by FDA for low stages of resistance [4]. Several studies have compared rTMS with electroconvulsivotherapy (ECT). ECT showed a higher efficacy in acute severe depression, psychotic depression and high level of treatment resistance. It is therefore considered as the gold standard therapy for patient in TRD with 60% to 90% rate of acute response but with side effects such as acute cognitive impairment, arrhythmias, and headaches. At the long course, this approach is not well adapted to chronic TRD as DBS might be.

Comparing to TMS and ECT, DBS is an invasive and still experimental treatment. Even if it is reversible, it requires a complex neurosurgical procedure with a risk of surgical complications such as infection, intracranial hemorrhage, lead erosion and migration. Thus, this treatment should be proposed to patients with a very high level of resistance. In an ethical point of view, as well as for scientific reason, this therapeutic procedure should be recommended under the supervision of an ethical committee within the frame of an experimental study [5].

Currently DBS in the subgenual cingulate gyrus, in the ventral capsule/ventral striatum, and in the nucleus accumbens (Nac) has showed encouraging results in the treatment of major depression [6–10].

Based on anatomical and functional arguments as well as clinical observations we investigated in the present paper the role of the Nac as a primary target for DBS.

### Material and methods

We based our review on electronic and manual literature search of MEDLINE, PUBMED and PSYCHINFO. The following terms were included:” Major Depressive Disorder”, “Deep Brain Stimulation” and “Nucleus accumbens”. Titles (and abstracts when available) were screened for relevance, leaving a list of 27 articles concerning the mechanisms of action of high frequency brain stimulation and 54 articles concerning the pathophysiology of MDD and the anatomophysiology of the Nac. Eventually we reported 5 studies using the Nac as a target for DBS.

### Results

#### Mechanisms of action of high frequency brain stimulation

##### An ablation-like effect?

Despite its demonstrated clinical efficiency in a variety of neuropsychiatric conditions, the therapeutic mechanisms of DBS remain unclear. Until the late 1980s, the neurosurgical treatment of choice for disabling drug-resistant tremor has been ventral intermediate nucleus (Vim) thalamotomy [11–13]. However, tremor recurred in about 20% of cases, and the adverse effects were not negligible [12]. DBS use began with Benabid et al’s work [14]. DBS showed comparable effects to Vim thalamotomy on tremor. Indeed 88% of the stimulated subjects showed complete relief from tremor or major improvement of their symptoms.

These observations led many investigators to conclude that DBS acts as a suppressor of neuronal activity [15, 16]. Several mechanisms have been proposed to explain this inhibition: 1) GABA-mediated suppression of neuronal activity around the stimulated electrode 2) elevation of potassium-current, 3) adenosine triphosphate (ATP) release and adenosine A1 receptor activation, and 4) enhancement of the rhythmicity and synchronous inhibition within and between afferent structures.

1. STN stimulation in humans could entail a suppression of neuronal activity around the stimulated electrode [17, 18]. Indeed, chemically induced thalamic neuronal inhibition by intra nuclear injection of muscimol (GABAergic agonist) appears effective in stopping tremor in the same way as DBS. These results suggest that GABA-mediated (thalamic) neuronal inhibition may represent a mechanism underlying the effectiveness of DBS [19].
2. On the other hand, Shin et al. [20] put forward the hypothesis that stimulation entails an elevation of potassium-current which decreases neuronal activity by the activation of an ion conductance resulting in membrane depolarization. In contradiction to previously exposed results, any influence of gabazine (GABAergic antagonist) application was observed, suggesting a synaptic-independent mechanism. In the same way in vitro patch-clamp techniques in rat STN slices [21] have showed that a brief tetanus produces a full blockade of STN activity, frequency dependent, which can be repeated without alteration, and not synaptically induced, since it was still observed in the presence of blockers of ionotropic GABA and glutamate receptors or in the presence of cobalt (voltage-gated calcium-channels blocker) [21]. Moreover stimulation entailed a blockage of persistent sodium-current and a reduction of calcium-mediated responses, suggesting that T- and L-type calcium-currents are transiently depressed by stimulation [22].
3. Another explanation could be that DBS causes adenosine triphosphate (ATP) release in astrocytes, resulting in accumulation of its metabolite, adenosine. Afterward adenosine A1 receptor activation could depress excitatory transmission, thus causing an inhibitory effect [23].
4. Eventually a study suggested that high-frequency Nac DBS suppresses pyramidal cell firing and enhances slow local field potential oscillations in the orbitofrontal cortex. This suppression might be mediated by antidromic activation of corticostriatal recurrent inhibition. Nac DBS may achieve therapeutic effects by enhancing rhythmicity and synchronous inhibition within and between afferent structures, thereby normalizing function of a neural circuit that presents aberrant activity in MDD [24].

##### A conflicting hypothesis: an activation-mediated effect of DBS

However the arguments in favor of a DBS ablation-like effect are still disputed. Thus, studies in parkinsonian primates and evidences from microdialysis studies in human subjects have demonstrated increased mean discharge rates of neurons in GPi during chronic stimulation in STN, and GPi stimulation in humans has been associated with a suppression of neuronal activity in the thalamus [25, 26]. Moreover a Positron Emission Tomography (PET) study showed that bilateral STN stimulation in patients with Parkinson’s disease increased blood flow significantly in the left midbrain while it decreased in cortical regions [26]. Functional magnetic resonance imaging (fMRI) also showed an increase in blood oxygen level-dependent signal in the subcortical regions ipsilateral to the stimulated nucleus [27].

Another argument in favour of the activation-hypothesis is that stimulation in external globus pallidus (GPe) has been demonstrated to improve bradykinesia [28], and yet a reduction in GPe output was supposed to worsen parkinsonian motor signs. Thus the improving effect of DBS in GPe might occur by a mechanism other than inactivation of neuronal activity in the stimulated area.

##### Toward a reunifying explanation

These data suggest that pattern of excitation or inhibition entailed by DBS is rather complex. Although arguments for increased output from the stimulated structure seem to conflict with the hypothesis that stimulation acts to inhibit neuronal activity, several hypotheses have been proposed to explain these paradoxical observations: 1) the activation of fiber pathways and 2) the independence of firing in the cell body and axon could resolve the apparently contradictory experimental results on the effects of DBS.

1. Activation of fiber pathways. Three classes of neurons can be affected by the stimulation: local cells, afferent inputs, and fibers of passage [29]. Local cells represent neurons that have their cell body very close to the electrode; afferent inputs represent neurons projecting axon terminals to the region of the electrode and whose axon terminals make synaptic connections with local cells; and fibers of passage represent neurons where both the cell body and axon terminals are far from the electrode, but for which the axonal process traces a path closely to the electrode. Currently it remains unclear which neuron class, or combination of neuron classes, are responsible for DBS therapeutic benefit. The effect of stimulation on cellular activity in the stimulated site could be increased or decreased, depending on the neurotransmitter of the afferent fibers. Thus, although high frequency stimulation may inhibit neurons via activation of inhibitory afferents, the output from the stimulated structure may be increased, resulting from the activation of axonal elements, as they generally possess a lower threshold for excitation than cell soma [30].
2. Another possible explanation could be that cell body firing could not accurately reflect the efferent output of stimulated neurons. This decoupling of somatic and axonal activity could explain the paradoxical experimental results [31].

Eventually the therapeutic mechanisms underlying high frequency DBS could most likely result from a combination of several phenomena, and may involve complex interactions and potential mechanisms of combined grey and white matter stimulation effects [16, 32]. Currently, there is a lack of consensus about the mechanism of action of DBS and more studies are still necessary to understand its therapeutic potential. Regardless of whether axons are activated or inhibited by high frequency DBS, defining the specific fiber tracts modulated by DBS could be a first step toward understanding its therapeutic mechanisms.

##### Diffusion-tensor imaging: applications for DBS

Diffusion-tensor imaging (DTI) is a non-invasive imaging technique that can be used to define axonal trajectories through white matter areas of the brain [33–35]. Johansen-Berg et al. [36] used probabilistic tractography to define the likely connectivity of cingulate regions stimulated by DBS. This technique provides statistical details on how likely a given pathway is to connect one brain region to another. Future applications appear for DTI in DBS use: probabilistic tractography could be used to statistically define the most pertinent connected anatomical regions [36]. DTI could also been used to predict the volume of tissue activated by DBS on a patient-specific basis [37]. Another application of DTI could predict the spatial extent of action potential generation in response to specific stimulation parameter settings [38]. Moreover correlation analyses could be performed with functional imaging data (fMRI and/or PET) to identify directly stimulated fiber populations, and their corresponding cortical and/or subcortical regions. If this kind of analysis was performed on enough patients it may be possible to statistically define the “actual target” of DBS. This information would be highly important in defining an optimal electrode implantation location, and motivate the use of patient-specific tractography in pre-operative surgical planning.

#### Pathophysiology of MDD

##### Functional neuroanatomy of MDD

Studies comparing depressed patients to healthy controls have showed anatomical and metabolic differences between groups in different regions. Frontal cortex has received the most attention in research on MDD. This focus makes sense, given the likely involvement of this region in MDD and its treatment.

Coffey et al. [39] reported a 7% volume reduction of the frontal lobe among 48 patients suffering from MDD comparing to 76 healthy volunteer subjects. Similar results were found in more recent works focused on orbito-frontal area with a reduction up to 32% compared with that of normal controls [40–42]. These OFC volumetric anomalies were closely associated to a less neuronal density [43, 44], and to cognitive disturbances [45].

Some vascular modifications [46], as well as a reduction of the density and size of the neurons were also highlighted in dorsolateral prefrontal cortex [44]. Moreover voxel- based morphometry (VBM) has been also used to quantify structural brain changes associated with MDD. It showed that gray matter was significantly reduced in dorsolateral and dorsomedial prefrontal cortex [47, 48].

These observations can be linked to functioning abnormalities observed in depressed subject with fMRI. Several studies have shown an increase of functional activity orbital and ventro-lateral areas of the prefrontal cortex [49–53]. This hyperactivity was correlated to the severity of depressive symptoms especially with sadness, with thought distortions, pessimism, guiltiness, self-devalorization, and with anxiety, which accompanies depressive syndrome [54, 55]. Increased activity of rostral anterior cingulate and dorsolateral prefrontal lobe has been found during effortful tasks [56–58]. It suggests a possibly compensatory activity to maintain task performance. On the other hand, a reduction of the functional activity of the dorso-lateral and dorso-median prefrontal cortex was reported in major depression [49, 50, 52, 59–61]. Moreover, an EEG study showed that within depressed persons, lower bilateral PFC activity predicts higher levels of rumination [62].

However, while the prefrontal cortex is undoubtedly involved in clinical aspects of MDD, it is unlikely that dysfunctions in this region can explain all the symptoms of this disorder. Indeed frontal regions are implicated in working memory, attention, impulse control and other aspects of executive function. Abnormalities in these cognitive domains are observed in MDD, but it could be argued that such symptoms do not represent the main part of the symptomatology in many patients. Indeed a striking observation is the extent to which abnormalities in reward and motivation domains are seen in MDD. For example, most depressed patients prominently exhibit a reduced ability to experience pleasure (anhedonia) and loss of motivation, as well as abnormalities in several neurovegetative functions such as appetite, sleep, energy level, and circadian rhythms 1. The brain’s reward regions could be at the heart of the MDD’s manifestations. Studies from the drug addiction field have identified the nucleus accumbens (Nac; part of the ventral striatum) and its dopaminergic inputs from the ventral tegmental area (VTA) of the midbrain, as the one of the most important anatomical substrates for drug reward as well as for natural rewards, such as food, sex, and social interactions [63].

##### The neurotransmitter traffic and neurogenesis

These forebrain networks are modulated by monoamine projections from midbrain and brainstem nuclei (dopamine from the ventral tegmental area (VTA), serotonin from the dorsal raphe located in the periaqueductal grey area, and noradrenaline from the locus coeruleus) [64]. All available antidepressant medications are based on serendipitous discoveries of the clinical efficacy of two classes of antidepressants more than 50 years ago. These tricyclic and monoamine oxidase inhibitor antidepressants were subsequently found to promote serotonin or noradrenaline function in the brain. Newer agents are more specific but have the same core mechanisms of action in promoting these monoamine neurotransmitters [65].

Glutamate is also involved in the physiopathology of the MDD: studies showed that systemic administration of a N-methyl-D-aspartate (NMDA) antagonist have an antidepressant effect by rodents exposed to a chronic stress, and weak dose of NMDA antagonist administration entails an improvement of mood by depressed patients [66]. Glutamate also plays a modulation role of DA release. Indeed, administration of NMDA antagonist in Nac entails an increase of DA release [67]. Moreover recent works on the NMDA receptor antagonist ketamine showed that this substance leads to rapid and relatively sustained antidepressant effects in patients with treatment-resistant MDD [68, 69]. Animal studies suggested that this antidepressant effect could be mediated by enhancing AMPA relative to NMDA throughput in critical neuronal circuits [70, 71].

Moreover brain-derived neurotrophic factor (BDNF) regulates many neuronal aspects including cell differentiation, cell survival, neurotransmission, and synaptic plasticity in the central nervous system [72]. Its function is mediated by its binding to specific receptors, such as the TrkB receptor and the pan [73] neurotrophin receptor (p75NTR). BDNF expression is closely regulated by neuronal activity. Localization of the TrkB receptor also increases at synaptic sites after neuronal activity. p75NTR is a low-affinity receptor of BDNF, and it can mediate neuronal apoptosis only when the Trk receptor is less or not active [74]. Notably, it is possible that alteration in the expression and/or function of BDNF in the central nervous system is involved in the pathophysiology of various brain diseases, including depression [72]. Indeed in mouse model a passive profile (chronically defeated, with a high level of immobility and low non-social exploration) was associated with a lower hippocampal BDNF level than in mice with active profile. Therefore, it has been proposed that MDD is associated with impaired neuronal plasticity and that antidepressant treatments promote several forms of neuronal plasticity, including neurogenesis, synaptogenesis and neuronal maturation and also increase BDNF activity [74].

##### Endocrinology

On the other hand, glucocorticoids, stress-induced steroid hormones, also putatively contribute to the pathophysiology of depression [72]. MDD has been associated with impaired mineralocorticoid receptor function [73] and raised cortisol level [75]. Interestingly, in addition to the reduction in BDNF levels due to increased glucocorticoid exposure, current reports demonstrate possible interactions between glucocorticoids and BDNF-mediated neuronal functions. Other steroid hormones are involved in numerous neuronal events including cell survival and synaptic plasticity. Kendall et al. [76] have shown the influence of sex hormones (testosterone and estrogen) on antidepressant-induced alterations in 5HT-2 receptor binding. Moreover, it has been proved that estrogen plays a role in the pathophysiology of Parkinson’s disease, Alzheimer’s disease, and mental illness, while serving to regulate BDNF expression and function [72]. Moreover the estrogen decrease may be a factor in both the pathogenesis of late-life depression of the postmenopausal state and in therapeutic response [77].

#### Nucleus accumbens and MDD: anatomical arguments

##### Anatomical reminder

The Nac belongs to a subcortical telencephalic and diencephalic set of nuclei: the basal ganglia. It is located immediately underneath the anterior limb of the internal capsule and covers a large area of the basal forebrain rostral to the anterior commissure. Medially adjacent to it is the diagonal band of Broca; laterally adjacent to it are claustrum and piriform cortex. The Nac is dorsally adjacent to the rostral extensions of the globus pallidus and the anterior limb of the internal capsule. Dorso- laterally, the Nac extends into the ventral putamen, dorso-medially into the ventral caudate (i.e the ventral striatum). The Nac is divided into two parts: a central core and a peripheral shell. The central core is associated with the extrapyramidal motor, whereas the peripheral shell is associated with the limbic system [78].

Within the Nac, information is transmitted from shell to core. Nac core receives a dense dopaminergic input from the ventral tegmental area (VTA) and the dorsal tier of the substantia nigra, but also serotoninergic and noradrenergic afferences of respectively the raphe nuclei and the locus coeruleus, which are themselves innervating hippocampus, amygdala and VTA. The Nac receives glutamatergic afferences from the hippocampus, the baso-lateral amygdala and the prefrontal cortex [79]. Its main efferents innervate the pallidum, striatum, mediodorsal thalamus, prefrontal cortex and mesolimbic dopaminergic areas.

Thus the Nac occupies a central position between limbic structures (basal ganglia, amygdala, and mediodorsal nucleus of thalamus) and cognitive structures as prefrontal cerebral cortex. This key position allows it to play an important role in control of locomotion, motivation and in reward processing. Since dopamine (DA) is a major transmitter in the Nac, a function of modulation on amygdaloid–basal ganglia– prefrontal cortex circuitry can be supposed.

##### Dopaminergic pathways

Dopaminergic mesolimbic and mesocortical systems are involved in hedonia and motivation, two important dimensions in clinical picture of MDD. Several lines of evidence implicate the mesolimbic DA system in the pathogenesis and treatment of MDD. The mesolimbic pathway sends projections from VTA to the limbic system via the Nac, the amygdala, and the hippocampus as well as to the medial prefrontal cortex. Dopaminergic neurons label environmental stimuli with appetitive value, predict rewards and motivating events and poor adaptation of the DA system may be involved in MDD [80].

##### Serotonin-Dopamine-Glutamate interactions in the nucleus accumbens

Social defeat stress in animals leads to alterations of the mesolimbic DA system, and in an interesting manner the behavioral changes in these animals were reset by chronic application of a selective serotonin reuptake inhibitor (SSRIs) [81]. SSRIs are efficacious in MDD because of their ability to increase serotonin neurotransmission. This increasing might result in a suppression of the firing activity of DA neurons [81]. It has been showed that glutamate release in the Nac is involved in depressed behaviour during the Porsolt swim test [82]. Glutamate interacts with serotonin in the Nac: indeed NMDA antagonist perfusion in Nac entails an increase of serotonin release [80, 83]. Beyond its key role from the anatomical point of view, there are functional evidences of the interest of the Nac as a target for DBS by depressed patients.

#### Nucleus accumbens and MDD: functional arguments

##### Implication of the nucleus accumbens in normal and abnormal reward processes

Depressed subjects show accentuation of the negative perceptions along with incapacity to feel pleasure during positive stimulations. The ventral striatum, and especially the Nac, is a central region for processing reward and pleasure information. Increases in Nac neuron activity and DA release are observed during experience of rewards. The ventral striatum shows abnormal activity following administration of dextroamphetamine in patients with MDD, compared with activity observed in healthy control subjects. Dextroamphetamine is a DA releaser and a DA reuptake inhibitor with secondary serotonergic- and noradrenergic-releasing effects. At safe doses (5–60 mg), dextroamphetamine reliably stimulates brain reward system sites and produces measurable, characteristic, and well-studied pleasurable effects such as euphoria and increased drive. These results suggest the presence of a hypersensitive response in the brain reward system of depressed patients, which may reflect a hypofunctional state [84]. Furthermore, a recent study suggests that the reward system basal ganglia dysfunction in MDD may affect reward processing [85]. Thus the Nac is a critical centre for the experience of reward and pleasure, and is dysfunctional in patients suffering from MDD9.

##### Implication of the nucleus accumbens in anhedonia and loss of motivation

One core symptom of MDD is anhedonia, characterized by a lack of reward- motivated behavior, associated with a decreased experience of pleasure or interest in previously enjoyed activities. Anhedonic symptoms are linked to reward responses in key nodes of the reward system. The Nac acts as a “motivation gateway” between limbic systems involved in emotion, and systems involved in motor control. Loss of motivation and anhedonia found in the MDD can be bound to abnormalities of the reward system (dopaminergic mesolimbic pathway, whose VTA and Nac are the main actors).

Indeed a study of Yadid et al. [86] showed that repeated stress infers an increase of the DA release in Nac by normal rats but not by model rats of MDD, while a neuroimaging study found attenuated Nac activation in response to positive words in depressed subjects [17, 87]. A negative correlation between anhedonic symptoms and Nac responses to positive stimuli in a monetary gains paradigm has also been measured. Additionally, a morphometric study suggests that anhedonia in MDD is negatively related to the Nac volume [88]. In contrast to a previous study [89], this association was shown to be specific to Nac and did not extend to other basal ganglia regions (i.e.: anterior caudate volume).

Moreover, depleting DA from the shell region of the Nac in rats severely impairs their ability to engage in reward-seeking in a drug self-administration paradigm [90]. The Nac implication in anhedonia phenomenon may be mediated by the transcription factor cAMP response element-binding protein (CREB), since it plays an important role in regulating mood. In rodents, increased CREB activity within the Nac produces MDD- like symptoms, including anhedonia, whereas disruption of CREB activity by expression of a dominant-negative CREB (mCREB, which acts as a CREB antagonist) has antidepressant-like effects. These studies support the hypothesis that disruption of CREB in the Nac influences motivation by facilitating reward and reducing depressive- like states such as anhedonia and dysphoria [91].

Thus, the Nac mediates motivational behavior related to obtaining rewards. The Nac seems to be a key target in the treatment of MDD since anhedonia is one of the key defining symptoms of the disorder [9].

##### The nucleus accumbens: at the centre of a circuit involved in MDD

**Figure 1:**
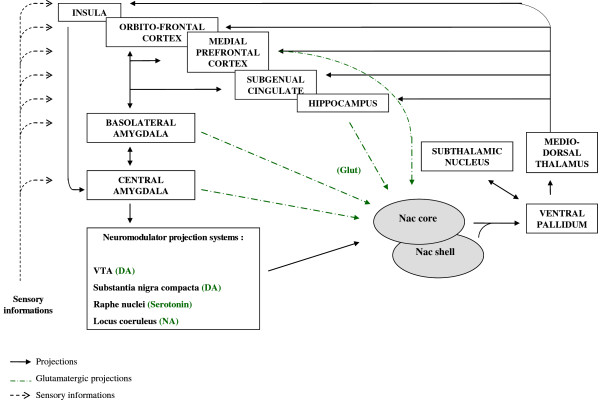
**Schematic of the limbic corticostriatal loop (from a schematic by Cardinal, 2006 showing key structures involved in reward process and anhedonia and loss of motivation observed in MDD.** DA: dopamine, Glut: glutamate, NA: noradrenalin, VTA: ventral tegmental area. The Nac seems to be at the centre of this circuitry: between the emotional system (amygdala, basal ganglia, insula, medial prefrontal cortex, orbitofrontal cortex, VTA), the cognitive system (anterior cingulate cortex) and motor control system (hippocampus).

The Nac is in connexion with VTA, the amygdala, orbitofrontal cortex, medial prefrontal cortex, motor regions such as the dorsal caudate and globus pallidus, and the hippocampus; and it turn indirectly projects to cortical regions including subgenual cingulate in Brodmann area 25 (Cg25) and medial prefrontal cortex, the ventral pallidum, the thalamus, and amygdala [91, 92] (see Figure 1). These connections of the Nac can be GABA-ergic or glutamatergic [47]. Evidence from neuroimaging, neuropathological, and lesion analysis studies show that many of these regions are implicated in identification of the emotional value of a stimulus, production and regulation of affective states, and automatic regulation of emotional responses [51].

The relationships between the medial prefrontal cortex, the rostral anterior cingulate cortex, and the amygdala appear to reflect an interaction between the self-referential processing and the negative emotional information processing and these interactions might be associated with depressive symptoms [93].

##### Altered functioning in patients with MDD

The Nac proved to be dysfunctional regarding rewarding stimuli in patients with MDD. In a functional imaging study of Epstein et al. [87], words with positive, negative or neutral value were proposed to depressed subjects versus control subjects. The results showed by depressed subjects a cortical activation significantly decreased at the level of the ventral striatum during presentation of words with positive value, correlated with the degree of loss of pleasure. These findings showed a dysfunction of the circuit of reward in the MDD underlined by neuronal substrates (dopaminergic pathways and Nac).

In another study [94] the authors combined fMRI with a dopaminergic probe (a dose of oral dextroamphetamine sulfate) to stimulate the brain reward system. Subjects with MDD showed a hypersensitive response to the rewarding effects of dextroamphetamine, with altered brain activation in striatal regions (including head of caudate, putamen and nucleus accumbens).

Taken together, these findings support a model of MDD including reward/motivational pathway dysfunction and suggest a crucial role of the Nac in the inability to experience pleasure or engage in rewarding activities.

#### Encouraging results

Because of its central role in reward circuitry and its dysfunctions regarding rewarding stimuli in patients with MDD the Nac appears to be a key structure in MDD treatment with DBS. There have been several reports to date on the effectiveness of stimulating of the Nac in *preclinical studies* and in patients suffering from MDD.

##### Pre clinical studies

Pre-clinical studies on animal models provide a better understanding of the mechanisms underlying the action of DBS. Hamani et al. [95] reviewed the findings with DBS on rodents for depression. Contrary to human studies where DBS is continuous, in preclinical studies, intermittent patterns have been investigated. In the study of Hamani et al. [96], DBS of the VMPFC and the NAC increased sucrose preference in a chronic mild unpredictable stress model, reflecting antidepressant like effects [97, 98]. Falowski et al. [99] studied DBS of the NAC in animal model of depression and showed an anti-anhedonic effect of DBS by an increase of the exploratory behavior and a decrease of an anxiety-like behavior. Dopamine and norepinephrine levels decrease and the length of apical and basilar dendrites in pyramidal cells increased supporting the hypothesis of a neuroplasticity induced by DBS. Interestingly, two other models of depression (helplessness model and tail suspension test) did not improve after DBS of VMPFC.

##### Clinical studies

The effectiveness of DBS in the ventral caudate nucleus in improving depressive symptoms was first reported in a case of primary treatment- resistant obsessive-compulsive disorder with concomitant MDD6. The patient underwent bilateral electrode stimulation with the deepest contact located in the Nac, and the superficial contact in the ventromedial caudate. DBS of the ventral caudate nucleus gradually improved depressive and anxiety symptoms until remission at six months, in parallel with an improvement in psychosocial functioning. Nevertheless, the psychiatric comorbidity in this patient limits extrapolation of these data to patients with clinical picture of only MDD. Afterwards, Aouizerate et al. (2009) tested DBS of the ventral striatum in two patients suffering from severely distressing and intractable forms of obsessive-compulsive disorder and MDD. Electrodes were implanted into the ventral striatum (including the head of the caudate nucleus and the Nac). Authors observed by the first subject an improvement in HDRS scores at six months after surgery; this improvement remaining up to fifteen months after surgery. A depressive symptoms worsening was first observed by the second subject over the first three months, afterwards a reduction of HDRS scores was observed, until remission, nine months after surgery. In another study, three patients suffering from extremely resistant forms of MDD were implanted with bilateral DBS electrodes in the Nac. Clinical ratings improved in all three patients when the stimulator was on, and worsened in all three patients when the stimulator was turned off. Effects were observable immediately, and no side effects occurred in any of the patients. Using FDG-PET, significant changes in brain metabolism as a function of the stimulation in fronto–striatal networks were observed [7]. *Bewernick et al.* in 2010 [8] studied the long- term effects of DBS in the Nac in a more important group of 10 patients. Twelve months following initiation of DBS treatment, five patients reached 50% reduction of the HDRS. The number of hedonic activities increased significantly by these patients. Interestingly, ratings of anxiety (Hamilton Anxiety Scale) were reduced in the whole group, markedly in the responder group. The positron emission tomography data revealed that Nac-DBS decreased metabolism in the subgenual cingulate and in prefrontal regions including orbital prefrontal cortex. A sustained effect of NAc DBS was shown in 5 out of 11 patients at four years of follow-up [100]*.*

The Nac is not the only target of DBS in human Treatment Resistant Depression (TRD). Five other areas have been stimulated. Anderson et al. [101] and Blomstedt et al. [102], reviewed these different targets and their efficacy. The most studied target is the subgenual cingulated gyrus (SCG). Mayberg et al. [103] showed an hyperactivity of this area in TRD patients compared to healthy subjects and Kennedy et al. [104] have shown that SCG might be considered as a biological marker of resistance. Three independent studies including 20 patients were realized with a mean reduction of of 52% after 1 year, at the HDRS score. Ventral capsule/ventral striatum (VC/VS) is another target of DBS, also used in refractory OCD [105]. A multicentre study driven with 17 patients showed a mean reduction at the HDRS score of 44%, after 1 year [10]. Two other targets have been proposed with only case reports: inferior thalamic peduncle because of antidepressant effect on preclinical model and lateral habenula because of hypermetabolism on depressed patients and on preclinical model. Recently, some promising results have been observed by targeting the foramen median bundle [106]. Currently, none of the targets has proved its efficacy in a randomized control trial.

### Discussion

Our review put forward the central role of the Nac in the physiopathology of MDD. Indeed anatomically and functionally the Nac is at the centre of the reward circuit involved in MDD. This structure is involved in both normal and abnormal reward processes and shows an altered functioning in patients with MDD. Moreover several clinical studies indicate the effectiveness of DBS in the ventral caudate nucleus in improving depressive symptoms.

Nevertheless, several limits should be noticed concerning the use of DBS applied to the Nac.

#### Parameters of DBS in Nac

In the human studies [8, 9] stimulating the NAC in MDD, parameters of stimulation were adapted at patient response. Although the pulse-width and frequency of stimulation remained stable (respectively 130Hz and 90micros), the voltage was adapted from 1,5 V up to 10 V with an increase of the voltage for non responders. The ideal settings to reach an antidepressant response are still unclear.

Pre clinical studies in animals were realized in order to improve the different parameters. In a large animal study using functional Magnetic Resonance Imagery (fMRI) during the stimulation of the NAC, the increase of the voltage from 3 to 5 V lead to an increase of the Blood Oxygenation Level Dependent (BOLD) signal in insula, thalamus and parahippocampal cortex and to a decrease in the cingulate and prefrontal cortex [99]. This study showed that functional response relies on effective level of currents. But, in a study on rodents testing the optimal setting using DBS in prefrontal ventromedial prefrontal cortex(VMPFC), Hamani et al. [96], showed that increasing the current up to 130HZ with a charge density of 100-130uA was associated with a worsening of the response. It implies that under and above a certain threshold, DBS could be ineffective.

At the present time, both shell and core were stimulated in DBS studies. It is difficult to determine which amelioration is due to the core’s stimulation and which one to the shell’s one. However it will be important in the future to determine how DBS acts on each structure. The role of Nac shell and core is quite well documented, but their role in MDD is still to determine more precisely.

Generally, the shell seems to constitute a limbic sector of the brain involved preferentially in motivational and emotional processes, whereas the core may comprise a striatal sector that plays a role predominantly in motor functions [107]. Pharmacological methods have shown differences between the Nac core and Nac shell in basal DA metabolism, and indicate that the core and shell DA innervations can be distinguished on the basis of response to both pharmacological and environmental challenges. These data were consistent with the hypothesis that the dopaminergic innervation of the Nac core is associated with the nigrostriatal system, while that of the Nac shell is related to the mesolimbic system [108]. Both microdialysis and voltammetry studies have shown that several drugs of abuse, such as cocaine, morphine, amphetamine and nicotine preferentially increase extracellular dopamine in the shell compared to the core of the NAC [109]. Excitotoxic lesions have also been used to investigate the functions of the Nac core and shell in animal models [110]. These results indicated a functional dissociation of subregions of the Nac in mediating amphetamine-potentiated conditioned reinforcement and locomotion. The shell appeared to be a critical site for stimulant effects underlying the enhancement of conditioned response and locomotion after intra-Nac injections of amphetamine, whereas the core seemed to be implicated in mechanisms underlying the expression of conditioned stimulus and unconditioned stimulus association. An animal study also showed that DBS of the Nac shell significantly attenuated the reinstatement of drug seeking precipitated by high cocaine doses whereas DBS of the dorsal striatum had no influence on cocaine reinstatement [111]. Since the shell seems to constitute a limbic sector of the brain involved preferentially in motivational and emotional processes, we tend to think that it will become a preferential target in depression treatment.

The issue of the long term antidepressant benefit of Nac DBS seems to be solved as showed in Bewerick et al’ studies [8, 100]. However, potential concerns regarding possible long-term habituation with stimulation of the Nac can be questioned. Intracranial self-stimulation has been largely studied in animals. In this behavioural paradigm, animals repeatedly press a lever to stimulate their own dopamine-releasing neurons electrically. Dopamine and GABA neurotransmission in the Nac play a role in this phenomenon; with robust changes in cell firing of many Nac units [112, 113]. Moreover a case of habituation has been reported by a subject with obsessive-compulsive disorder [114]. This subject exhibited intra-operatively an asymmetric smile and acute positive emotional change with DBS in Nac and anterior limb of the internal capsule region. Chronic DBS resulted in habituation of the smile response. This habituation was characterized by the loss in intensity of the facial response during successive testing over the initial 3 months following surgery. At 6 months and a year following the operative procedure, longer washout periods in the “DBS-off” condition of 16 hours and 2–4 weeks respectively (on the right side), failed to restore the smile or affective response. These findings imply potential habituation and changes in the neural chemistry (possibly neuroplasticity) induced by chronic DBS.

## Conclusions

To conclude there are strong preclinical and clinical arguments to consider that DBS of the Nac may be an effective strategy in MDD treatment. Recently DBS to brain structures mediating reward and motivation processes has showed encouraging results. It is also interesting to notice few side effects of this treatment have been observed. Nonetheless the therapeutic mechanisms of DBS must be still determined; these findings might offer a focused and novel approach to treating refractory MDD with a favorable efficacy to side effect profile.
